# Neural Vision Restoration in Ophthalmology

**DOI:** 10.1007/s10439-026-03989-y

**Published:** 2026-02-13

**Authors:** Jainam Shah, Sachin Pathuri, Joshua Ong, Raena Greenbaum, Nikolas Melkumyan, Ryung Lee, Kimia Rezaei, Andrew D. Parsons, Jason Zheng, Karl Golnik, Andrew G. Lee

**Affiliations:** 1 Albert Einstein College of Medicine, Bronx, NY USA; 2 Creighton University School of Medicine, Phoenix, AZ USA; 3 Department of Ophthalmology and Visual Sciences, University of Michigan Kellogg Eye Center, Ann Arbor, MI USA; 4 Department of Internal Medicine, University at Buffalo, Buffalo, NY USA; 5 University of California Riverside School of Medicine, Riverside, CA USA; 6 California University Science and Medicine, Colton, CA USA; 7 Department of Neurology, Barrow Neurological Institute, Phoenix, AZ USA; 8 Center for Space Medicine, Baylor College of Medicine, Houston, TX USA; 9 Department of Ophthalmology, Blanton Eye Institute, Houston Methodist Hospital, Houston, TX USA; 10 The Houston Methodist Research Institute, Houston Methodist Hospital, Houston, TX USA; 11 Departments of Ophthalmology, Neurology, and Neurosurgery, Weill Cornell Medicine, New York, NY USA; 12 Department of Ophthalmology, University of Texas Medical Branch, Galveston, TX USA; 13 University of Texas MD Anderson Cancer Center, Houston, TX USA; 14 Texas A&M College of Medicine, Bryan, TX USA; 15 Department of Ophthalmology, The University of Iowa Hospitals and Clinics, Iowa City, IA USA

**Keywords:** Neural interfaces, Visual neuroprostheses, Brain-computer interfaces, Retinal prostheses, Cortical stimulation, Artificial intelligence, Machine learning, Adaptive stimulation, Closed-loop systems, Neuroplasticity, Visual rehabilitation, Ophthalmology

## Abstract

Neural vision restoration is a rapidly advancing discipline at the intersection of neuroscience, bioengineering, and ophthalmology. This review synthesizes emerging strategies to restore visual perception through retinal prostheses, optic nerve and thalamic implants, cortical brain-computer interfaces (BCIs), optogenetics, and non-invasive stimulation. Although initial experiments have demonstrated primitive visual abilities such as light perception and motion detection, artificial vision remains cognitively demanding and fundamentally different  from natural vision. Advances in artificial intelligence and machine learning may enable adaptive, closed-loop systems that optimize stimulation, enhance low-light vision, and integrate environmental inputs for more intelligible percepts. At the same time, a growing understanding of neural plasticity, cortical remapping, and perceptual learning highlights the need for multidisciplinary strategies in visual rehabilitation. Ethical and regulatory concerns, including informed consent, data protection, neural enhancement, and equitable access, remain central to responsible implementation. The potential of BCIs to bypass the eye entirely, and of neuroprosthetics to be used in spaceflight, disaster response, or military medicine, expands the applications of vision restoration beyond blindness alone. Bridging technological, clinical, and ethical strategies in this review outlines the challenges and opportunities that define the future of neural ophthalmology. Ultimately, restoring sight will require not only functioning hardware, but systems compatible with the reorganized brain and the lived experience of visual loss.

## Introduction

The human visual system is one of the most complex yet least regenerative neural systems following injury or neurodegeneration. Vision loss from conditions such as glaucoma, retinitis pigmentosa (RP), and optic neuropathies is often irreversible due to the central nervous system’s limited capacity for repair [1]. Traditional therapies have targeted the eye using optical aids, in some cases enabling basic visual perception. However, these approaches rely on residual ocular structures and offer minimal benefit in advanced disease or optic nerve damage [1].

In response to these limitations, neural vision restoration has emerged as an alternative strategy that bypasses damaged visual pathways through direct neural stimulation rather than attempting to repair dysfunctional  ocular tissue. These approaches introduce artificial patterns of neural activity at different levels of the visual system, with the goal of eliciting percepts that the brain can learn to interpret as visual information. Unlike natural vision, which depends on photoreceptor-driven transduction and highly structured retinal signaling, neural vision restoration relies on externally generated stimulation that substitutes for lost biological input. Current neural vision restoration strategies include retinal implants, optic nerve implants, thalamic implants, cortical implants, brain-computer interfaces (BCIs), optogenetic reactivation of surviving neurons, and computational encoding methods that transform visual scenes into simplified stimulation patterns. Across these modalities, functional vision depends not only on device performance but also on the brain’s capacity to adapt to non-physiologic input through perceptual learning and neuroplasticity.

Because artificial stimulation cannot fully replicate the spatial and temporal complexity of native retinal signaling described above, many current neural vision restoration systems remain limited by low-resolution percepts, variable outcomes, and extensive training requirements to achieve functional utility [2]. Additionally, many neural vision restoration technologies remain in early clinical stages, with limited long-term safety data [2]. A multidisciplinary effort is required to address these issues, as bioengineers must optimize hardware, clinicians must minimize complications, and ethicists must address questions surrounding user autonomy, agency, and authorship of action during long-term neural interfacing in real-world trials [2, 3]. Overcoming these challenges is critical for translating experimental systems into viable, long-term vision restoration solutions.

This review provides an ophthalmology-focused synthesis of contemporary neural vision restoration strategies, spanning device-based neural interfaces, perceptual and cognitive adaptation to artificial vision, computational image encoding, and the ethical and regulatory considerations that accompany clinical translation and downstream applications. Rather than presenting these technologies as isolated advances, this review examines how engineering constraints, neurobiological plasticity, and clinical context jointly shape functional outcomes in patients with severe vision loss. Specifically, it analyzes neural interface platforms targeting different levels of the visual pathway, mechanisms of phosphene perception and perceptual learning, the role of artificial intelligence (AI) in visual scene encoding, and the translational barriers that influence the durability, scalability, and equity of various approaches. Ethical, legal, and regulatory considerations are addressed with an emphasis on BCIs, which introduce distinct challenges related to long-term neural integration, autonomy, and privacy. By situating these discussions within their appropriate clinical context, the review aims to clarify both the promise and the limitations of neural vision restoration. Ultimately, the ability to restore vision without a functioning eye is not solely a technical challenge. It will require coordinated advances in neuroengineering, personalized stimulation strategies, and responsible clinical translation to deliver equitable, feasible, and durable visual recovery. Integrating these approaches introduces the potential to restore meaningful visual function, enabling individuals with severe vision loss to read text, recognize faces, and navigate their environments more independently [2].

## Methods and Literature Search

A structured narrative review of the literature was conducted by querying the Scopus and PubMed databases for peer-reviewed literature relating to vision restoration via neural interfaces. The search strategies utilized a combination of free-text terms and Medical Subject Headings (MeSH) in an effort to achieve both thematic breadth and specificity. Boolean operators were used for the principal terms such as “neural prostheses,” “retinal implants,” “cortical visual prostheses,” “optic nerve stimulation,” “brain-computer interface,” and “visual neuroprostheses,” with AND/OR combinations to identify areas of overlap between device engineering, clinical implementation, and neuroplasticity. The remaining keywords were “retinitis pigmentosa,” “age-related macular degeneration,” “optogenetics,” “closed-loop prosthetics,” “AI in ophthalmology,” and “ethical implications of BCIs.” Truncation operators (e.g., *) were used to catch any variations in terminology (e.g., “neuroprosthetic” vs. “neuroprostheses”).

The search was restricted to publications published between 1998 and May 2025. Because this review is narrative rather than systematic, inclusion was based on thematic relevance to device efficacy, perceptual outcomes, surgical technique, neuroadaptive strategies, and regulatory approval landmarks. Both English and non-English abstracts were screened, including publications in Chinese, Japanese, German, French, Spanish, Korean, and Portuguese, as screening non-English abstracts helps reduce language bias and aligns with recommended practices in review methodology [4]. High-quality systematic reviews and landmark trials were manually referenced for additional sources. Clinical trials were cross-referenced on ClinicalTrials.gov for current or recently completed studies involving retinal, thalamic, optic nerve, or cortical implants.

Priority was given to studies that involved AI, machine learning, or neuroplastic training in device optimization. In cases of more than one review on a given topic, the most recent and methodologically rigorous review was selected. Tables were constructed to present an overview of clinical trials, device features, perceptual standards, and regulatory status.

## Neural Interface Approaches to Vision Restoration

Neural interface technologies for vision restoration differ primarily by the anatomical site at which stimulation is delivered within the visual pathway, with each target imposing distinct constraints on surgical access, signal fidelity, perceptual outcomes, and clinical applicability. Accordingly, this section is organized by anatomical level of intervention, progressing from retinal interfaces to optic nerve and lateral geniculate nucleus (LGN) stimulation, and finally to direct cortical visual prostheses. This structure reflects how increasingly downstream targets bypass larger portions of the native visual system and expand potential indications, while introducing new technical and neurophysiologic challenges. Non-invasive and adjunctive neuromodulation approaches are addressed separately as rehabilitative strategies that may complement implant-based systems.

### Retinal Level Interfaces: Implants, Prosthetics, and Optogenetic Strategies

Retinal prostheses offer a way to restore vision in individuals suffering from degenerative retinal diseases such as age-related macular degeneration (AMD) and RP, which can cause irreversible vision loss [5]. In the healthy retina, outer retinal photoreceptors begin phototransduction and subsequent signaling to retinal ganglion cells (RGCs), whose axons form the optic nerve. Although photoreceptors are lost in RP and related disorders, much of the inner retina remains structurally and functionally intact [6]. The inner retina consists of four layers: the inner nuclear layer, which contains bipolar, horizontal, and amacrine cells; the inner plexiform layer, where these neurons synapse with RGCs; the ganglion cell layer, which contains RGC cell bodies; and the nerve fiber layer, which is formed by RGC axons [5]. Retinal implants bypass damaged photoreceptors by electrically stimulating surviving inner retinal neurons to generate visual percepts.

Epiretinal implants are arrays of microelectrodes placed onto the inner retinal surface to stimulate retinal neurons. A peripheral camera captures visual input, which is converted to electrical signals and passed through a periocular device to the implanted chip, which then forwards the information to the remaining inner retinal neurons [7]. This yields a qualitatively different visual experience from normal perception and requires cognitive adaptation from the patient. In 2013, the United States Food and Drug Administration (FDA) approved ARGUS II, the first commercial retinal prosthetic in the U.S., which was developed by Second Sight Medical Products [8–12]. ARGUS II improved basic visual functions (e.g., object localization, motion detection, reading letters) and had a modestly low complication rate because the epiretinal implantation surgery was similar to other routine retinal surgeries [9]. However, limited visual acuity (max 20/1260) and the invasiveness of the device prevented it from being used in AMD patients (a much larger portion of the population than those with RP only), and the device was discontinued in 2019 [13]. The IRIS II, approved for Conformité-Européenne (CE) marking in 2016, was also withdrawn in 2018. Although early clinical testing demonstrated improvements in high-contrast object localization, motion detection, and picture recognition, subsequent reports with the IRIS II noted significant issues with device durability [14, 15]. Multiple IRIS II implants experienced functional decline within 9–12 months due to halted device operation. Several serious adverse events were reported, including hypotony from sclerotomy leakage, vitreoretinal traction related to vitreoschisis at the tack site, and persistent ocular pain [16]. Because of these reliability concerns, Pixium Vision discontinued IRIS II development and redirected efforts toward its more stable PRIMA subretinal platform. Additional epiretinal systems with advanced features in development include EPI-RET 3, IMIE 256, and POLYRETINA (Table 1).

**Table 1:** Key clinical trials in retinal technologies for vision restoration (2006 − 2025)

Device	Approach	Company	Indication	Efficacy	Status
ARGUS II[13]	Epiretinal	Second Sight Medical Products	RP	Best visual acuity 20/1260	FDA-approved in 2013, discontinued in 2019 due to funding issues
IRIS II[29]	Epiretinal	Pixium Vision	RP	Elicited photosenses	CE-approved in 2016, discontinued in 2018 due to funding issues
EPI-RET 3[30]	Epiretinal	RWTH Aachen University	RP	Elicited photosenses	Phase 1/2a
NR600	Epiretinal	Nano Retina	RP	No results posted	Terminated in 2024 due to funding issues
POLYRETINA	Epiretinal	École Polytechnique Fédérale de Lausanne	RP	No results posted	Animal studies
IMIE 256	Epiretinal	Golden Eye Bionic and IntelliMicro Medical	RP	Elicited photosenses	Phase 1/2a
Alpha IMS / AMS[31, 32]	Subretinal	Retina Implant AG	RP	Best visual acuity 20/546	CE-approved in 2013 and 2016, discontinued in 2019 due to funding issues
PRIMA[22, 23]	Subretinal	Pixium Vision	AMD	Best visual acuity 20/460	Phase 3 ongoing
HARP4K	Subretinal	Iridium Medical Technology Company	RP & AMD	No results posted	Animal studies
BRIP	Subretinal	Bionic Eye Technologies Inc. and Visus Technology	RP	No results posted	Animal studies
44-channel Suprachoroidal Retinal Prosthesis[33]	Suprachoroidal	Bionic Vision Australia	RP	Elicited photosenses	Phase 1/2a
49-channel suprachoroidal-transretinal stimulation (STS)[34]	Suprachoroidal	Osaka University	RP, Stargardt disease	Elicited photosenses	Phase 1/2a
Phoenix	Suprachoroidal	Bionics Institute	RP	No results posted	Animal studies
CPCB-RPE1[35]	Subretinal – stem cell implant	Regenerative Patch Technologies	AMD, Stargardt disease	> 5-letter gain	Phase 1/2a
OpCT-001	Subretinal – stem cell injection	BlueRock Therapeutics	Primary photoreceptor disease	No results posted	Phase 1/2a ongoing
DSP-3077	Subretinal – stem cell injection	Sumitomo Pharma	RP	No results posted	Phase 1/2a ongoing
MCO-010[28]	Optogenetic	Nanoscope Therapeutics Inc.	RP, Stargardt disease	Improved best-corrected visual acuity (BCVA)	Phase 2b
RTx-015	Optogenetic	Ray Therapeutics	RP	No results posted	Phase 1 ongoing
Ray RST-001	Optogenetic	AbbVie	RP	No results posted	Phase 2a ongoing
GS030	Optogenetic	GenSight Biologics	RP	No results posted	Phase 1/2a ongoing

Retinal Implant AG developed two subretinal prostheses, the Alpha IMS and Alpha AMS, which were CE-marked in Europe in 2013 and 2016, respectively, for the rehabilitation of RP patients. These devices used an implanted photodiode electrode array for the direct conversion of light to electrical impulses, without the need for external cameras or cables to replace missing photoreceptors [17]. This maintains the link between eye movements and visual stimuli, and clinical trials demonstrated improvement in light perception, object identification, and other activities of daily living [13, 18]. However, the implantation procedure is technically more challenging than ARGUS II due to adhesions between the retina and retinal pigment epithelium (RPE), potentially requiring a posterior retinotomy [19]. Adverse events were more common but remained manageable, and the safety profile was considered to be clinically acceptable [20]. Although Alpha devices produced superior long-term results and improved visual acuity (up to 20/546) compared to ARGUS II, Alpha devices were not FDA-approved, had limited use, and were discontinued in 2019 for economic and regulatory reasons [18].

Current research aims to improve retinal implants by reducing the pixel size to ensure higher visual acuity and greater utility. However, as pixel dimensions decrease, each pixel delivers less charge per unit area, meaning that smaller pixels will require more intense stimulation to achieve effective activation of inner retinal neurons [21]. The Pixium Vision Photovoltaic Retinal Implant (PRIMA), a subretinal photodiode array, is an implant that offers visual acuity up to 20/460 and targets central vision loss in AMD with minimal impact on peripheral vision [22, 23]. It is the only optoelectronic implant in clinical trials specifically for AMD. PRIMA uses specialized augmented reality (AR) glasses equipped with a forward-facing camera and a near-infrared (NIR) projection system; these glasses capture visual scenes, process them in real time, and project 880-nm NIR patterns onto the implant, where they are converted into electrical stimulation of inner retinal neurons [22, 23]. Earlier versions employed opaque virtual-reality–style goggles to isolate prosthetic vision, whereas newer transparent AR glasses allow patients to simultaneously use natural peripheral vision along with central prosthetic input [22]. Because the glasses deliver encoded NIR patterns directly onto the implant, optimal performance depends on accurate subretinal placement beneath the central area of geographic atrophy, ensuring that stimulation reaches the remaining inner retinal neurons. Positioning this implant in the atrophic zone enables restoration of prosthetic central vision without affecting residual peripheral visual acuity [23]. A three-dimensional honeycomb array is also being researched as a means of enhancing electric field penetration with smaller pixels [24]. Other subretinal implants in development include HARP4K and the Boston Retinal Implant Project (Table 1).

Other novel strategies under investigation include suprachoroidal implants and stem cell therapies. Suprachoroidal implants, located between the choroid and sclera, avoid the vitreous cavity and present a lower risk of damaging the retina. However, they are farther away from target cells, which means higher stimulation power is required, and this distance limits visual improvement compared to epi- or subretinal implants. Despite this, their favorable safety profile supports further development. Ongoing technologies include the Generation 2 44-channel implant, Phoenix 99 Bionic Eye, and suprachoroidal-transretinal stimulation (Table 1). Stem cell therapies aim to replace damaged RPE or photoreceptors. Key limitations include poor integration and limited survival of transplanted cell suspensions, which has prompted biomaterial-based approaches such as monolayer scaffolds [25]. Potential complications include tumor growth, inflammation, and retinal detachment [26]. Active projects to establish new stem cell therapies include CPCB-RPE1 (RPE implantation), OpCT-001 (photoreceptor precursor injection), and DSP-3077 (retinal sheet injection).

Gene therapy has also been proposed as a means of introducing non-native light-sensitive proteins into surviving retinal neurons for RP patients, which would induce photosensitivity at earlier disease stages than those suited for prostheses [27]. Goggles, similar to certain optoelectronic devices, are used to amplify environmental light to stimulate these proteins. This method may offer greater visual acuity than implants but carries the risk of inflammation and toxicity. In 2024, MCO-010 became the first optogenetic treatment to show positive clinical trial results for vision restoration in RP, and an FDA biologics license application has been in progress [28]. Other trials currently investigating gene therapy include RTx-015, Ray RST-001, and GS030.

While retinal prosthetics, implants, and gene therapies have potential for visual restoration in RP and AMD patients without significant adverse events, their broader adoption remains constrained by several persistent limitations, including limited visual acuity improvements, invasive interventions, therapeutic risk, high developmental costs, regulatory hurdles, and a limited eligible patient population. Ongoing efforts must aim to improve outcomes, lower costs, and expand indications for these innovations to become clinically and economically feasible.

### Optic Nerve and Thalamic Targets for Vision Restoration

Because retinal-based approaches depend on the presence of viable inner retinal neurons, alternative strategies have explored stimulation at more proximal locations within the visual pathway, including the optic nerve and thalamus. One such approach involves direct stimulation of the optic nerve with implanted electrodes. The artificial vision by direct optic nerve electrode (AV-DONE) device, which was trialed from 2007 to 2009 in three RP patients, was positioned over the optic disc to evoke phosphenes, which are visual percepts that vary in shape and color and are often yellow [36, 37]. Although localized percepts were achieved, their irregularity limited the quality of vision. Computational modeling has attempted to address this issue by optimizing electrode arrays to improve positioning and signal fidelity [38]. The OpticSELINE intraneural array was demonstrated to selectively stimulate optic nerve fibers and produce distinct cortical activity patterns in animal studies, a promising result for ongoing efforts to create non-overlapping phosphenes [39, 40].

**Fig. 1:**
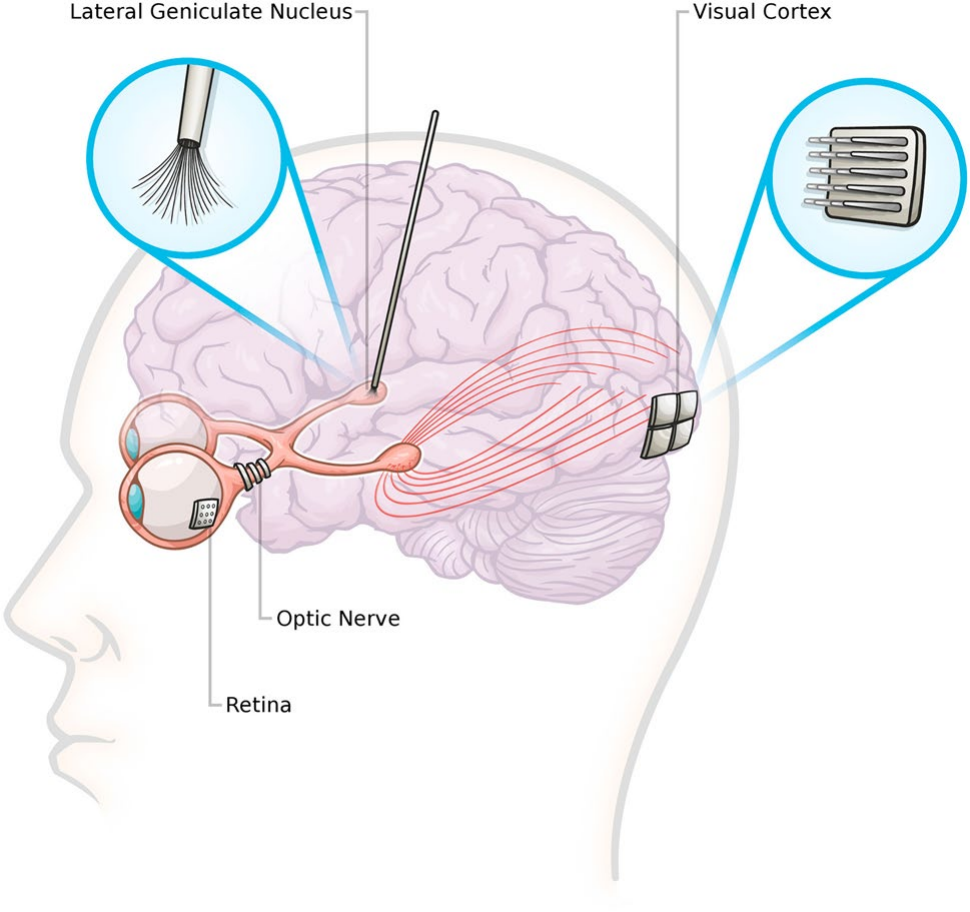
Anatomical diagram illustrating electrode placement targeting the LGN within the thalamus for visual prosthetic stimulation. Figure and legend reproduced from (Lewis et al., 2015) [45] under the Creative Commons Attribution–NonCommercial–NoDerivatives 3.0 Unported License (https://creativecommons.org/licenses/by-nc-nd/3.0/). PMID: 25446438

The LGN, a thalamic relay between the retina and the visual cortex, is also being investigated as a prosthetic target in individuals with retinal degeneration or optic nerve damage. Electrical stimulation models suggest that LGN stimulation may restore vision in patients who have damaged retinal input from glaucoma or optic neuropathy [41]. Previously considered surgically inaccessible, recent developments in deep brain stimulation now allow electrodes to be implanted in the LGN of animals [42]. In neuropathic disease, the RGCs or optic nerve are damaged, but the downstream visual pathways remain structurally intact. By directly stimulating LGN neurons, the dysfunctional anterior structures are bypassed, restoring visual perception through intact thalamocortical circuits (Fig. 1) [42, 43]. Current research remains focused on the optimizing stimulation parameters, including amplitude, duration, and frequency, for effective LGN stimulation [43]. Optogenetic stimulation of the LGN has also been shown to evoke artificial visual percepts, further highlighting how thalamocortical circuits can support visual reconstruction even in the absence of normal retinal signaling [44].

### Visual Cortex Stimulation – Direct Brain Stimulation Approach

Devices in development also target the visual cortex of the occipital lobe, which represents the most downstream point of intervention, as the pre-geniculate and geniculate structures are bypassed. One advantage of this cortical approach is that its efficacy is not limited by ocular diseases, such as retinal atrophy, corneal or lens opacities, or optic nerve damage. These devices can thus be useful for individuals with vision loss due to glaucoma, diabetic retinopathy, optic nerve trauma, ocular cancer, or ocular trauma. Notable models include the Orion Visual Cortex Prosthetics system (next-generation Argus), the Cortical Vision Neuroprosthesis for the Blind (CORTIVIS), the Intracortical Visual Prosthesis, and the Blindsight brain implant (Table 2). These systems use external cameras and microelectrode arrays implanted on or within the visual cortex. In 2024, the FDA designated Blindsight as a breakthrough device, a noteworthy development given the complex regulatory landscape for BCIs (Table 2) [46]. Preliminary data suggest that partial visual restoration is feasible, but continued development in microelectronics and biocompatibility is necessary to improve both safety and function.

**Table 2:** Key clinical trials in optic nerve and visual cortex–stimulating technologies for vision restoration (2009–2025)

Device	Approach	Company	Indication	Efficacy	Status
AV-DONE [38]	Optic nerve	Nidek Co.	RP	Elicited photosenses	Phase 1/2a
OpticSELINE	Optic nerve	Swiss Federal Institute of Technology in Lausanne (EPFL)	RP	No results posted	Animal studies
Orion	Visual cortex	Cortigent, Inc. (formerly Second Sight Medical Products)	Bilateral blindness	No results posted	Phase 1 ongoing
CORTIVIS	Visual cortex	Universidad Miguel Hernández de Elche	Bilateral blindness	No results posted	Phase 1/2a ongoing
ICVP	Visual cortex	Sigenics, Inc. and the Illinois Institute of Technology (IIT)	Bilateral blindness	No results posted	Phase 1 ongoing
Blindsight	Visual cortex	Neuralink	Bilateral blindness	No results posted	Phase 1 ongoing

### Non-Invasive and Adjunctive Neural Stimulation Approaches

In contrast to implant-based systems that deliver direct visual input, non-invasive neuromodulation and vision augmentation may offer adjunctive rehabilitation strategies. Techniques such as transcranial magnetic stimulation (TMS), transcranial direct current stimulation (tDCS), ultrasound stimulation, and AR aim to restore residual vision without surgery through cortical plasticity and perceptual retraining. Anodal tDCS to the visual cortex has been shown to enhance contrast sensitivity and increase visual evoked potentials in both amblyopic and neurotypical adults [47]. The addition of tDCS to vision restoration therapy accelerates stimulus detection and supports long-term field recovery in post-stroke homonymous hemianopia [48]. Effects are task-specific: perceptual gains occur within the first month, but visual field border shifts develop more gradually. In subacute stroke, tDCS-augmented therapy improved field sensitivity by 36.7%, compared to a 10.7% improvement with standard rehabilitation [49]. These findings suggest that tDCS can prime cortical networks to remain more sensitive to repeated training stimuli.

Other non-invasive techniques, including TMS and low-frequency ultrasound, offer additional benefits. Focused ultrasound can activate the visual cortex regardless of whether an intact optic nerve is present, and it can outperform light-based stimulation in response time [50]. TMS has been used to experimentally modulate visual processing, but its rehabilitative role is less well-established. Recent vision-specific reviews emphasize that while non-invasive brain stimulation shows promise in treating conditions such as amblyopia, age-related vision loss, and post-stroke visual field defects, clinical outcomes remain heterogeneous. They are highly dependent on baseline visual function, stimulation parameters, cortical target, and task context, underscoring the need for individualized protocols and larger, rigorously controlled trials [51]. In particular, contemporary syntheses highlight that reported benefits are often variable, task-limited, and most robust when non-invasive stimulation is combined with structured visual perceptual learning, supporting a rehabilitative rather than prosthetic model of intervention [52]. Collectively, these tools offer potentially scalable rehabilitation options for patients who remain ineligible for invasive implants, but they remain early-stage and exploratory in their current clinical application.

## The Neuroscience of Synthetic Vision: Cortical Processing and Adaptive Mechanisms

### Phosphene Encoding, Perceptual Learning, and Cognitive Integration

Across these diverse neural interface strategies, the immediate output of stimulation converges on a shared perceptual phenomenon: the generation of phosphenes. Rather than serving as direct visual analogs, phosphenes act as stable but abstract sensory cues whose functional meaning emerges through cortical association, memory, and multisensory integration. The resulting perceptual experience varies with the location of stimulation, device settings, and user adaptation. In an ethnographic study, Argus II and IRIS II users described their vision as “flickering lights” or “Eiffel Tower lights,” and developed a personalized “lexicon of flashes” through prolonged training involving memory, touch, and auditory integration [12].

**Fig. 2:**
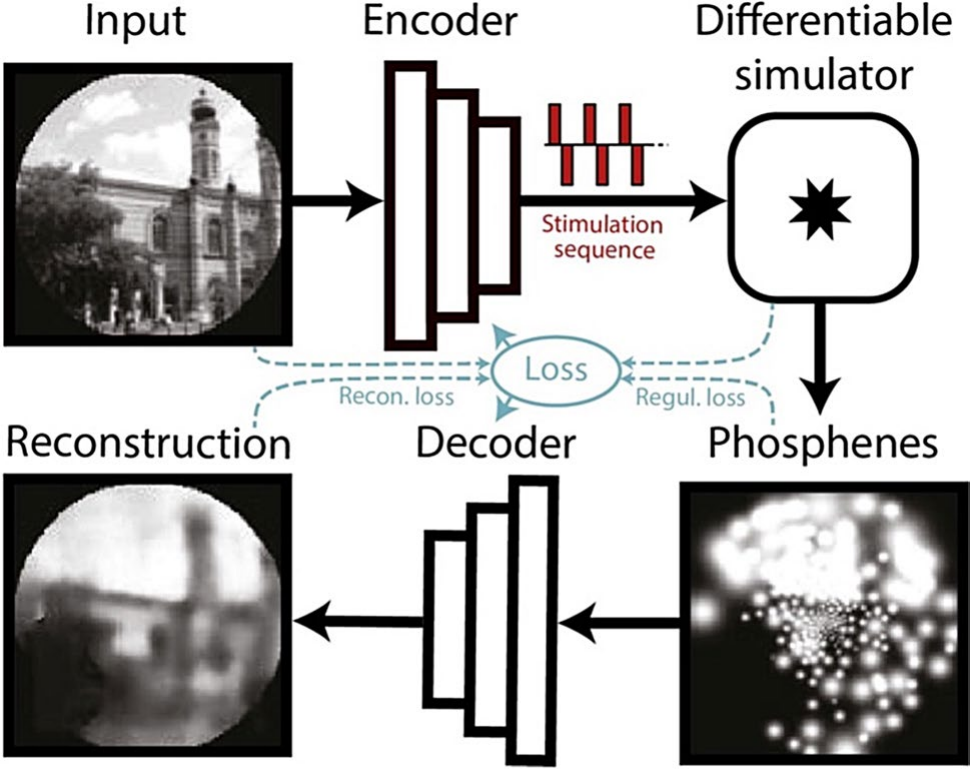
Diagram of phosphene interpretation. Figure and legend reproduced from van der Grinten et al. [55] under the Creative Commons Attribution 4.0 International License (https://creativecommons.org/licenses/by/4.0/). PMID: 38386406

Phosphenes produced by epiretinal devices are distorted by the spread of current and unsolicited axonal activity [53]. However, through perceptual learning, patients have been able to reconstruct images, and learn both letter recognition and navigation (Fig. 2) [54, 55]. Beyond the retina, stimulation of the optic nerve and LGN has also been shown to evoke structured phosphenes that can be interpreted by the visual system. Spiral cuff electrodes have created retinotopically organized percepts, and non-invasive optic nerve stimulation has improved visual fields in glaucoma patients [56, 57]. Optogenetic stimulation of the LGN has also been successful, yielding behaviorally relevant percepts in animal models [44]. Importantly, these observations reflect differences in the anatomical site of stimulation rather than the temporal pattern by which electrodes are activated, which represents a separate and independent determinant of phosphene structure.

 The manner in which electrodes are activated plays a critical role in phosphene perception. While simultaneous multi-electrode stimulation produces diffuse and spatially fragmented phosphenes, dynamic sequential stimulation yields coherent forms such as letters without prior training [58]. Importantly, this distinction refers to temporal encoding strategies rather than differences in the anatomical site of stimulation. V1 simulation models confirm that cortical anatomy imposes constraints on perception under simultaneous activation, supporting the use of sequential and current-steered strategies [59]. Temporal synchrony is also critical; synchronous stimulation enhances reading accuracy and object recognition, while asynchronous input causes these abilities to deteriorate [60]. The success of prosthetic vision will depend on spatial and temporal fidelity, neuroplastic adaptation, and patient engagement. Motivation, cognitive retraining, and support from neuro-ophthalmologists and rehabilitation specialists will be crucial in producing meaningful vision.

### Neural Plasticity and Long-Term Adaptation in Artificial Vision

Because phosphenes do not carry inherent visual meaning, their transformation into usable vision depends on the brain’s capacity to reorganize, relearn, and integrate artificial sensory input over time. The effectiveness of neural interfaces therefore reflects not only device design but also the ability of the visual system to adapt following long-term deprivation. Neural plasticity, whether adaptive or maladaptive, shapes how artificial vision is processed and applied. Long-term adaptation involves cortical reorganization, cross-modal reassignment, and perceptual retraining, and it is the biological basis for success or failure in prosthetic vision. Neural remodeling following photoreceptor loss occurs at multiple levels of the visual system, including the retina, thalamus, and visual cortex. Caravaca-Rodriguez et al. proposed a three-phase model in RP: glutamate receptor changes (Phase 1), gliosis and deafferentation (Phase 2), and neuronal migration with microneuroma formation (Phase 3) [61]. As synaptic remodeling, gliosis, and neuronal disorganization occur, stimulation thresholds increase, and retinal signal encoding becomes less precise, leading to degraded and inconsistent afferent input to downstream visual pathways. As a result, visual deprivation is not confined to retinal circuitry but propagates forward, reshaping thalamocortical signaling and cortical receptive field organization. Because glaucoma primarily disrupts retinal ganglion cell output while leaving cortical architecture structurally intact, it provides a clinically relevant model for examining post-retinal plasticity driven by chronic deafferentation. Contrary to previous beliefs, functional magnetic resonance imaging (fMRI) mapping in glaucoma patients shows that V1–V3 undergo localized receptive field shifts linked to visual field loss [62]. In congenital achromatopsia, V1 remains constant, but V3 adapts by expanding parafoveal sampling, suggesting hierarchical plasticity [63]. Collectively, these neuroimaging and clinical findings suggest that cortical reorganization following visual loss is both region-specific and experience-dependent, with higher visual areas retaining greater adaptive flexibility. For neural vision restoration, this plasticity represents a double-edged constraint: it may enable long-term perceptual learning, but it also implies that prosthetic input must be carefully aligned with the reorganized cortical landscape to achieve stable and meaningful visual function.

When visual deprivation is early, prolonged, or complete, this adaptive process extends beyond reweighting within the visual hierarchy and instead recruits non-visual sensory systems. For example, in congenital and early-onset blindness, cross-modal reallocation allows hearing and touch input to recruit the visual cortex, reflecting a shift from visual processing toward broader sensory integration.. Lewis et al. showed strong occipital responses to non-visual stimuli that were consistent with full cortical repurposing [64]. Animal and human studies confirm that early blindness promotes rerouting of the senses, although these adjustments may compromise visual reintegration [65]. This spectrum of plasticity has direct clinical relevance, as post-injury rehabilitation closely reflects this possibility. Interventions following traumatic brain injury, including prism correction and oculomotor function retraining, are promising but underused [66]. Home-based vision restoration therapy improved patient-reported outcomes, with modest gains in objective outcomes [67]. These findings redefine vision restoration as reconnecting, rather than replacing, and they emphasize the need for therapies that are congruent with the reorganized visual network of the brain.

## AI and Machine Learning in Neural Vision Restoration

### AI-Driven Visual Encoding for Neural Prosthetic Systems

Because phosphenes do not intrinsically encode visual meaning and must be actively interpreted through learning and cortical adaptation, the transformation of real-world scenes into usable prosthetic input represents a central challenge in neural vision restoration. As a result, computational approaches that selectively filter and encode visual information have become an important area of investigation. AI is increasingly being used in visual neuroprosthetics to convert complex visual scenes into interpretable patterns of neural stimulation that are compatible with current electrode array constraints. For example, Sanchez-Garcia et al. used a dual-channel segmentation framework that combined object detection and edge extraction to improve navigation and object recognition in prosthetic vision simulation [68]. Convolutional neural networks filtered out noise and emphasized structural elements, rendering schematic presentations more interpretable for users.

Building on these feature-extraction approaches, other researchers have employed biologically inspired attention models to further prioritize task-relevant visual information. One approach used graph-based saliency with GrabCut optimization to highlight foreground objects, followed by pixel enhancement techniques that improved object recognition across environments [69]. Reinforcement learning was applied in Task Optimized Phosphene Vision (TOPhos), which trained agents in dynamic tasks to generate compressed, task-specific stimulation patterns that outperformed traditional methods [70]. In this context, the term ‘agents’ refer to software-based reinforcement learning controllers operating entirely within simulated environments, where their performance serves as a proxy for how effectively different phosphene-encoding strategies support task-guided behavior [70]. Although these findings come from simulations rather than human subjects, they provide a proof-of-concept that adaptive, task-dependent encoding may ultimately enhance the functional value of neuroprosthetic stimulation. Another step advancing this integration was made by Neurolight, a deep-learning interface for intracortical stimulation, which blended ganglion cell modeling, semantic segmentation, and neural encoding to offer adaptive stimulation to the visual cortex [71]. As an end-to-end system, Neurolight provides an integrated open-loop pipeline from video input to cortical stimulation rather than a true closed-loop device, since it does not incorporate real-time neural or behavioral feedback. Nevertheless, its modular architecture demonstrates how modern deep-learning pipelines can be directly coupled to cortical microelectrode arrays, providing a critical translational framework for future systems that may incorporate real-time feedback for closed-loop control.

AI also enables targeted improvements in areas such as facial recognition by informing how visual information should be selectively prioritized under severe resolution constraints. For example, statistical region-of-interest magnification based on face-detection algorithms significantly improved recognition performance compared with direct resolution reduction, particularly when contextual features such as hair or head shape were preserved, and it reduced gender-related recognition bias [72]. Although these approaches rely on deterministic computer vision rather than adaptive learning, they exemplify AI-adjacent pre-processing strategies that shape which visual features are presented to downstream encoding and stimulation pipelines. More broadly, AI technologies have been classified into three domains: computer vision pre-processing, biophysical simulation of retinal response, and deep learning for task-adaptive encoding, providing a useful framework for organizing computational strategies across different stages of prosthetic vision pipelines [73]. Hybrid models combining biological models with adaptive AI appear especially promising, responding to user needs and environmental complexity. These tools are becoming increasingly crucial in balancing camera input and cortical perception, offering interpretable, scalable output that enhances function and user experience as resolution improves.

### Machine Learning for Personalized Visual Stimulation

Building on AI-driven scene encoding, machine learning (ML) approaches attempt to shift the focus from optimizing visual representations in general to adapting stimulation strategies at the level of the individual user. Whereas earlier AI frameworks primarily transform visual input into task-relevant phosphene patterns, ML can potentially enable stimulation parameters to be iteratively adjusted based on patient-specific neural responses, behavioral feedback, and learning trajectories. In this context, ML has the potential to offer a direct route to customized visual stimulation by supporting adaptive, data-driven refinement of prosthetic output over time.

Unlike static encoding techniques, ML-based systems may offer adaptive strategies that can potentially support closed-loop operation when paired with real-time feedback, allowing stimulation parameters to be modified in response to user performance or task demands. This approach would optimize perceptual resolution and support long-term integration through adaptive encoding and presentation of visual information to the brain. Although most current AI systems remain open-loop, deep-learning models demonstrate how closed-loop capability may eventually be achieved by learning to adjust stimulation based on behavioral feedback. Beyeler and Sanchez-Garcia’s “smart bionic eye” model uses user input and virtual reality training to refine scene-specific overlays, such as semantic segmentation and object search filters [74]. Such AI-driven innovations are continuously maintained and updated to allow real-time visual processing when navigating and conducting social tasks. Related modeling efforts include AI-based BCIs dynamically adjusting stimulation parameters based on patient activity patterns and learning [75]. Hybrid models that combine neural networks with traditional filters (e.g., Kalman, alpha-beta) enhance predictive accuracy and are able to adjust their settings to compensate for fatigue, neuroplasticity changes, or interface dynamics [76].

Clinical translation of ML-enabled visual prosthetics will require robust, transparent, and responsive systems capable of working with sparse or imbalanced data. Although AI and ML can excel at ophthalmic diagnostics, relatively few models have been incorporated into vision prosthetics [77]. Solutions to encourage broader adoption involve overcoming regulatory barriers and accommodating rare diseases or early-stage implants. AI has also been used to forecast treatment response in retinal diseases, such as diabetic retinopathy and AMD, a predictive capability that could guide visual stimulation based on cortical plasticity and patient goals [78]. This type of advancement brings prosthetic vision closer to personalized, real-world application.

## Ethical, Legal, and Societal Challenges of BCIs in Vision Restoration

### Informed Consent and Patient Autonomy

Despite their potential for vision restoration, BCIs present ethical, legal, and societal challenges that must be addressed. Because many BCI-based vision restoration systems remain investigational, informed consent is complicated by uncertainty surrounding long-term risks, particularly those related to neuroplastic adaptation [79]. It has been easier to define, describe, and characterize short-term risks related to BCIs, such as intraoperative complications and device setup, within the informed consent process. Meanwhile, long-term risks, such as adverse neuroplasticity, especially in younger patients, are speculative and must be investigated [80]. As a result, researchers may be unable to adequately counsel patients on potential future complications due to the absence of long-term longitudinal data. This highlights the necessity of developing a framework that acknowledges current gaps in long-term data and allows for reconsenting patients as new evidence emerges to ensure that ethical standards are maintained and patient autonomy is respected.

### Data Privacy and Cybersecurity Risks

Beyond physical and neurobiological risks, BCIs introduce ethical challenges related to the continuous collection and transmission of neural data. The ownership, storage, and control of neural data collected by BCIs are complex issues that have yet to be well-defined. A 2020 survey of BCI researchers found that 58% believe that patients should be able to access their raw neural data at the conclusion of a study. However, the majority of these researchers also believed that there should be safeguards in place to prevent individuals from donating or selling these data [81]. Currently, there are no unified privacy frameworks for all BCIs. This deficiency could introduce the possibility of third-party companies accessing sensitive brain data without the patient’s explicit consent or understanding [82]. Legislation mandating that companies use neural data only for its originally intended purpose and require its disposal after that purpose is fulfilled has been introduced in California, Minnesota, and Colorado; however, no comparable framework currently exists at the national level [83–85].

Due to a lack of privacy guidelines, BCIs do present cybersecurity risks and could potentially be hacked. While some risks are common among other medical devices as well, the cybersecurity concerns of BCIs can be argued to be more severe due to their interaction with the brain. Risks such as data theft, loss, or corruption as well as physiological takeover are all cybersecurity threats that may be associated with BCIs and other medical devices. However, BCIs may introduce additional cybersecurity threats such as unwanted movement, undesired brain stimulation, and incorrect vision processing and speech synthesis [86]. Although some BCI functions can be disabled in software, Schroder et al. note that deeper failures, such as corrupted firmware, unsafe stimulation tables, or compromised onboard controllers, cannot be corrected through software alone [86]. Because implanted BCIs are designed to operate for 12–15 years without physical access, recovery depends on secure wireless update pathways with integrity checks and rollback mechanisms; if these safeguards fail, surgical intervention may be the only option [87]. Therefore, additional risks arise from the need for invasive recovery and repair procedures. These factors emphasize the importance for robust security measures and risk management strategies as BCIs continue to evolve and become more widely deployed.

As BCIs increasingly rely on AI-mediated signal processing and wireless updates, security strategies have begun to incorporate AI-based protections. AI-assisted task automation can reduce vulnerabilities associated with human errors, especially in access control, anomaly detection, and real-time threat response [88]. Compared to traditional security architectures, AI has the potential to anticipate and respond to breaches in advance and has shown greater accuracy in detecting intrusions [89, 90]. However, reliance on AI introduces several risks of its own. If one part of the system is hacked, the entire framework can be compromised, and AI models are still susceptible to failure or adversarial attacks [91]. Moreover, these systems require personalization, financial investment, and technical expertise to use properly [92]. While AI offers powerful technologies for protecting BCI systems, its implementation must be carefully planned.

### Regulatory and Legal Challenges

**Fig. 3:**
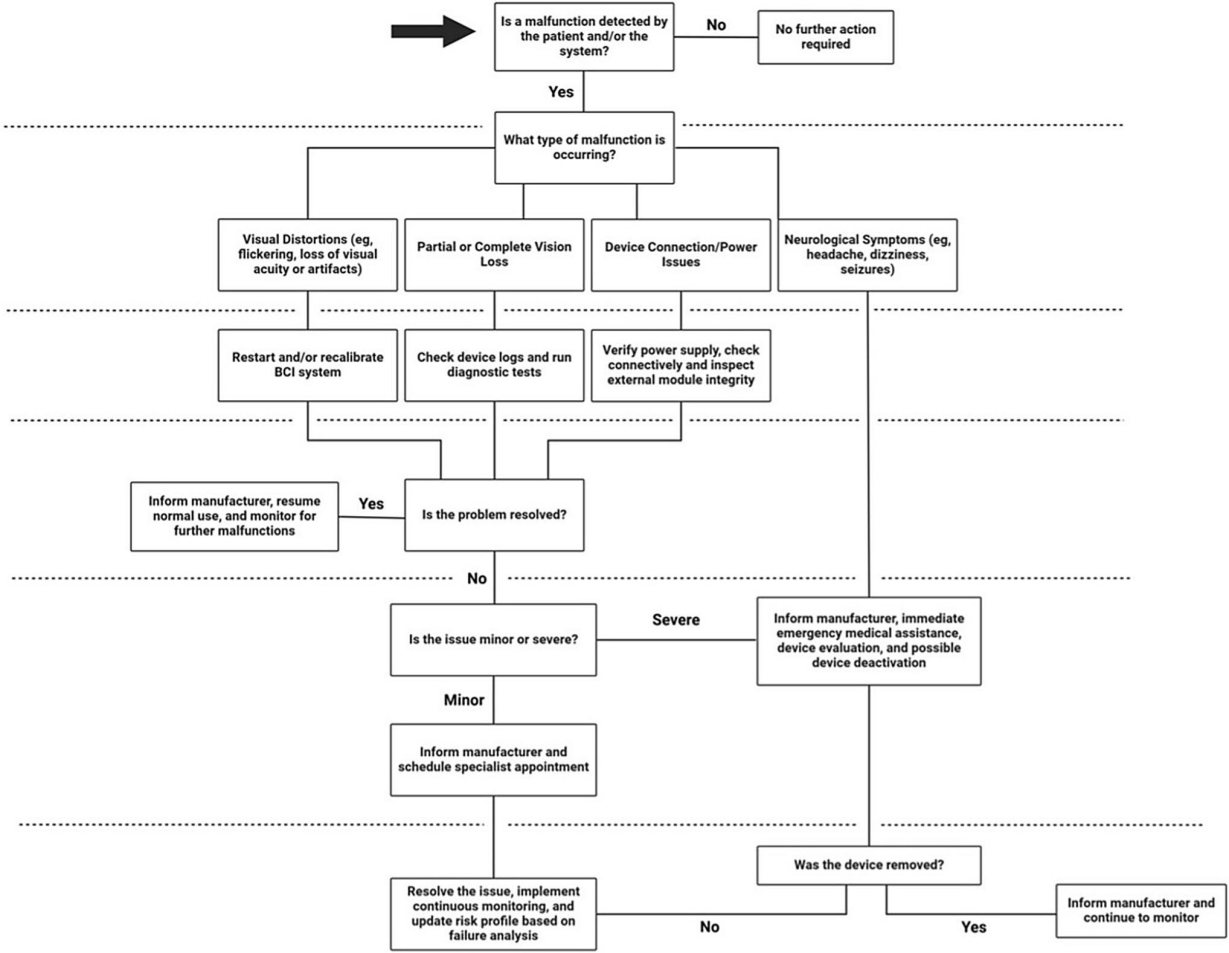
Malfunctioning brain–computer interface flowchart (created using Lucidchart.com)

These technical and data-related risks of BCIs raise downstream regulatory questions regarding responsibility, oversight, and long-term accountability. There are typically multiple stakeholders upon whom the responsibility for a BCI malfunction may fall (Fig. 3). These include the manufacturers of the BCIs, who are responsible for meeting regulatory requirements for safety and efficacy; the healthcare providers, who are responsible for implantation, maintenance, and patient monitoring; and regulatory bodies, such as the FDA, which must approve devices and perform post-market BCI surveillance. Experts have argued that AI-driven BCIs should be regulated differently from other medical devices [93]. AI-driven software is based on an evolving learning pattern; therefore, the traditional FDA and IRB models of one-time approval for ongoing or continuous use would not be appropriate. Instead, regulators must develop new mechanisms to both evaluate and longitudinally audit AI-driven software to ensure it continues to meet ethical standards.

### Neural Augmentation vs. Restoration: Ethical Boundaries

Ethical concerns extend beyond safety and governance to questions of purpose and intent. Experts have argued that the primary purpose of BCIs in the context of visual prostheses should be to restore functional vision to individuals who have previously lost it. Some have even proposed the development of an “artificial vision” rooted in artificial intelligence-based scene understanding rather than solely aiming to restore natural vision [74]. Ethical dilemmas arise when discussing the possibility of enhancing human vision beyond typical human capabilities. The use of BCIs for such enhancements could undermine the ethical principles of justice and create inequalities if enhancements are prioritized over restorative applications, particularly for individuals with disabilities. Empirical studies of visual prostheses show that simply defining what constitutes “meaningful restoration” varies widely across blind and sighted individuals, and current devices rarely achieve full functional recovery [94, 95]. Because restorative outcomes already differ substantially among users, shifting research or clinical resources toward enhancement risks deepening inequities for those still struggling to obtain even basic functional gains.

Additionally, concerns have been raised about patient autonomy with the alteration of cognitive functions by BCIs [96]. Enhancing vision could cause a change in the way people experience reality; users of sensory neuroprostheses have reported shifts in perception, emotional response, and self-understanding during early adaptation, raising questions about how neurotechnology may affect one’s sense of authenticity and agency [97, 98]. This in turn may lead to a change in their perceptions, thoughts, emotions, or sense of self, potentially compromising their autonomy.. Several authors have warned that, once enhancement-capable neurotechnologies exist, competitive academic or workplace environments may create implicit pressure to adopt them to avoid disadvantage, a form of “soft coercion” documented in other neurotechnology domains [99, 100]. This may lead to ethical dilemmas surrounding the concept of autonomy as individuals may opt to adopt BCI enhancements to maintain social status or acceptance rather than by their own autonomous choice [101].

### The Psychological Impact of Neural Vision Restoration

Beyond ethical and regulatory considerations, BCI-based neural vision restoration also carries important psychological implications for patients adapting to artificial vision. Depression is common among people with visual impairment, with studies reporting that roughly 10–45% of visually impaired individuals experience clinically significant depressive symptoms [102]. As BCIs become more common, integrating psychiatric and psychological support into vision rehabilitation programs may be essential. Depression has been associated with poorer adaptation to vision aids and slower reading speed improvements during visual rehabilitation [103]. Qualitative work in low-vision rehabilitation programs has shown that depressive symptoms can limit the ability to acquire new device-use skills, reduce session engagement, and impair problem-solving during training, leading to poorer functional outcomes even when the technology is effective [104]. Although these findings come from standard low-vision rehabilitation rather than BCIs, they highlight a clinically important pattern: mood symptoms are associated with impaired cognitive flexibility, persistence, and learning capacity, which are necessary for adapting to a novel sensory modality.

Because early BCI-based vision restoration will demand similar high cognitive load adaptation and repeated training sessions, untreated depression could produce analogous barriers to skill acquisition and real-world functional use. Addressing underlying depression may improve a patient’s BCI learning by improving attentiveness, flexibility, and motivation. Implementing such strategies requires a multidisciplinary framework involving ophthalmologists and psychiatrists to screen for depressive symptoms before training begins and to incorporate mental health metrics and psychotherapy, such as cognitive behavioral therapy, into personalized BCI rehabilitation protocols. Emerging work also suggests that BCIs themselves may eventually play a role in monitoring or modulating emotional states. Passive BCIs have been used to estimate mood and detect depressive patterns, and closed-loop systems paired with neuromodulation have shown measurable improvements in treatment response [105]. However, emotional regulation differs fundamentally from detecting discrete neural events: affective states involve distributed networks and sustained rhythms, making it challenging to identify stable neural features for real-time classification or intervention. These technical constraints underscore that psychological support cannot be offloaded to the device and must remain an integral component of early BCI rehabilitation.

### Socioeconomic Barriers and Accessibility

**Fig. 4:**
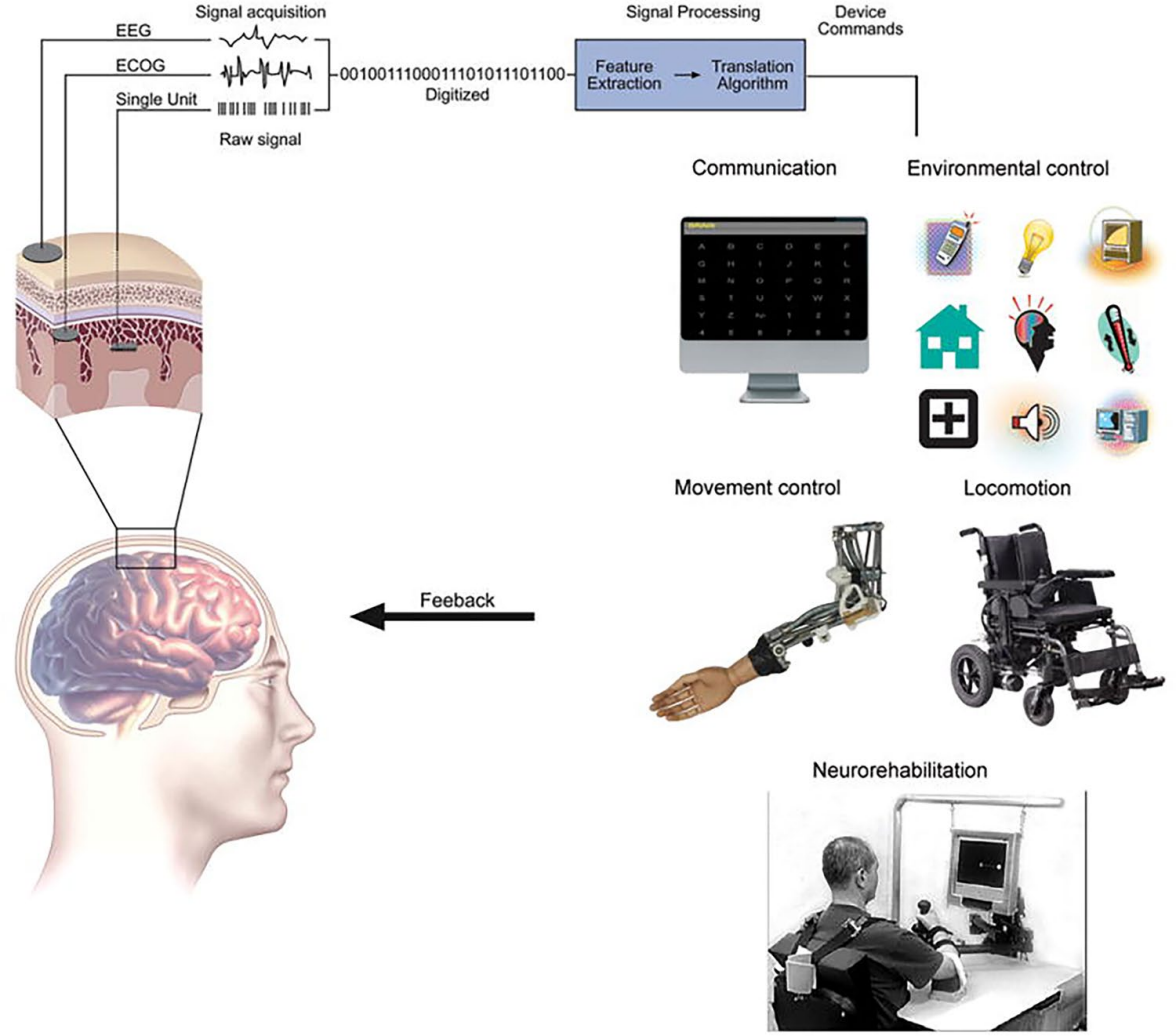
Simplified overview of a BCI system used across communication, environmental control, movement control, locomotion, and neurorehabilitation applications. Neural activity may be recorded using non-invasive EEG, electrocorticography (ECoG), or single-unit electrodes. Raw signals are amplified, digitized, and processed through feature extraction and translation algorithms to generate device commands. Outputs can control external devices such as computers, smart-home interfaces, prosthetic limbs, wheelchairs, or rehabilitation systems, with real-time feedback enabling adaptive user training. Figure and legend reproduced from Kawala-Sterniuk et al., 2021 [106] under the Creative Commons Attribution 4.0 International License (https://creativecommons.org/licenses/by/4.0/). PMID: 33401571

Without deliberate attention to accessibility, BCI-based neural vision restoration may risk violating principles of distributive justice by concentrating benefits among high-resource populations. To prevent inequities and make BCIs available to developing countries and underserved populations, several steps must be taken. First, there must be innovations focused on cost reduction, portability, and usability. While early in development, this focus has been reflected in studies describing the development of low-cost electroencephalogram (EEG)-based BCIs using off-the-shelf components (Fig. 4) [106]. Along with advancements in physical hardware, the creation of open-source solutions may promote broader use and adaptation in underserved communities. An example of such software today is the Blink-To-Live eye-based communication system designed to assist individuals with speech impairments by using four key eye gestures (left, right, up, and blink) to encode over 60 daily life commands [107]. The software and source code for this system are currently available on GitHub in an open-source format that allows it to be independent of specific hardware and be customizable to meet the user’s needs. However, cost remains a major barrier, as neuroprostheses are frequently described as disproportionately more expensive than traditional rehabilitation and require clinically and economically viable models for widespread dissemination [108–110].

Unequal affordability directly contributes to unequal access, with multiple authors noting that neuroprostheses may create new social inequalities if the devices remain inaccessible to lower-resource users or health systems [108, 109, 111]. With the hardware and software in place, there must also be a focus on providing training and educational resources for local healthcare providers and technicians. This need is supported by evidence that rehabilitation for neuroprostheses can be strenuous, resource-intensive, and time-consuming, often requiring extensive training that imposes physical, emotional, and financial burdens on users and families [99, 110]. Individuals without stable support systems or access to specialized rehabilitation services may face lower device success rates, further widening disparities. In the era of telemedicine and remote support, there is existing infrastructure that can provide ongoing support and training to individuals in remote areas with device setup, troubleshooting, and patient management. This infrastructure can be expanded with the development of certification programs and partnerships with local institutions. Finally, because neuroprostheses are considered scarce resources that must be distributed in a fair manner, equitable allocation frameworks will be needed to prevent widening gaps in access as BCIs become more clinically viable [97, 100].

## Emerging Technologies and Translational Challenges in Neural Vision Restoration

### Future Directions in Neural Vision Prosthetics

**Fig. 5:**
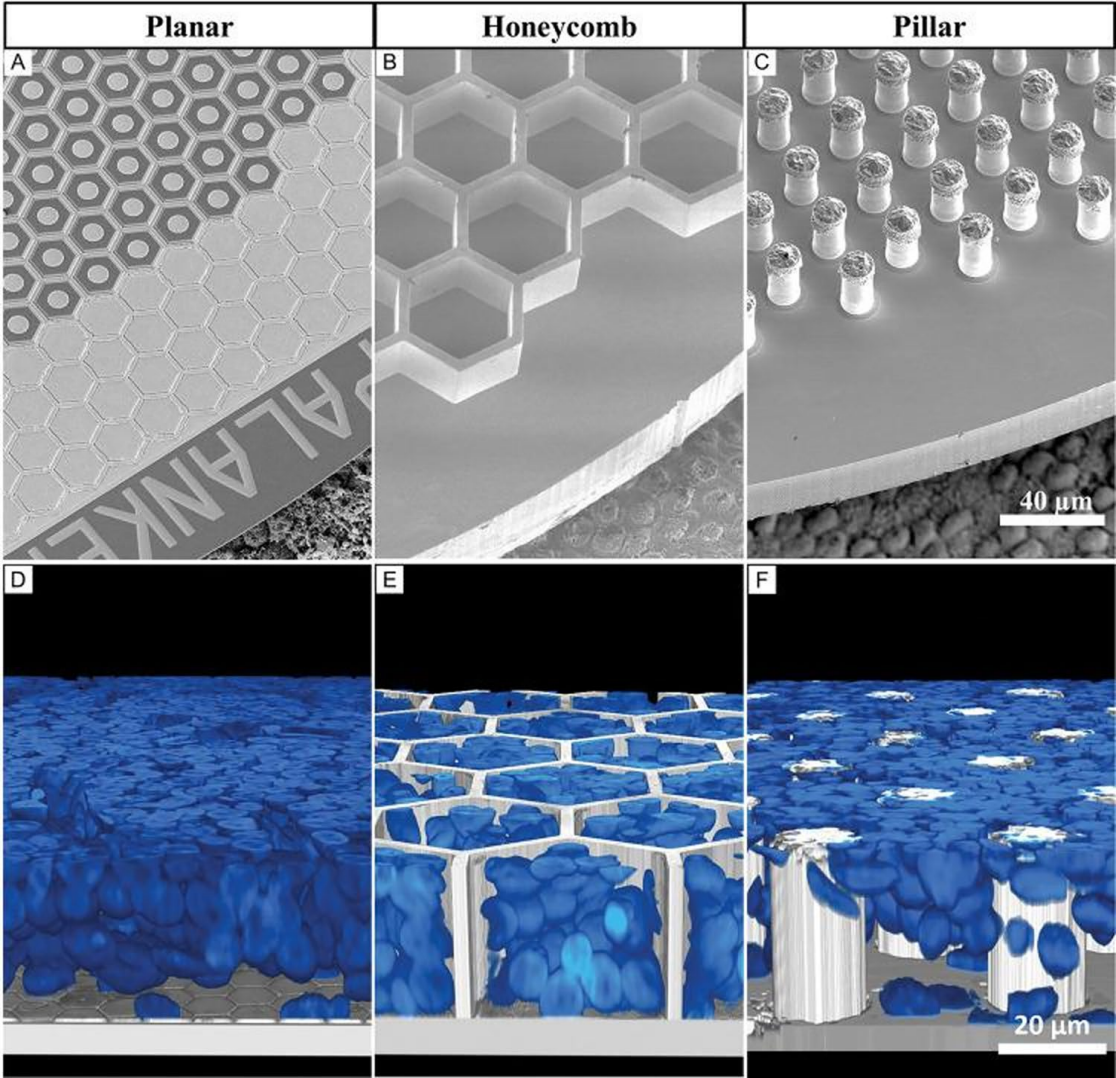
Scanning electron microscopy and confocal imaging of implant of three implant geometries—Planar (**A**, **D**), Honeycomb (**B**, **E**), and Pillar (**C**, **F**). All implants were fabricated in silicon and coated with titanium, with the pillar array additionally electroplated with gold. Confocal renderings show DAPI-stained retinal cell nuclei interacting with the implants six weeks post-implantation, demonstrating distinct patterns of inner nuclear layer (INL) integration. Figure and legend reproduced from Bhuckory et al., 2024 under the Creative Commons Attribution-No Derivatives 4.0 International License (https://creativecommons.org/licenses/by-nd/4.0/) [112]. PMID: 38659843

The long-term success of neural vision prosthetics will depend on their ability to dynamically co-adapt to users’ evolving neural and behavioral states. Current limitations in spatial acuity, interindividual variability, and long-term interface stability are being addressed through advances in materials science, neurobiology, and systems engineering. Despite these advances, current systems remain constrained by electrode density, perceptual variability across users, training burden, and uncertain long-term interface durability. In response to these constraints, emerging device architectures are shifting toward modular designs that permit iterative hardware optimization without complete system replacement. Subretinal prostheses, for example, are increasingly modular at the level of implant geometry and pixel architecture. In rodents, photovoltaic implants were safely upgraded *in situ* to smaller-pixel (22 µm) arrays for planar, honeycomb, and pillar geometries (Fig. 5) [112]. Although these studies were conducted in preclinical models, they establish a proof of concept for modular implant strategies that could ultimately enable staged upgrades or personalization in human patients as device performance and fabrication technologies improve. These modular hardware designs parallel ongoing efforts in stimulation control, with both approaches aimed at enabling adaptive systems rather than fixed stimulation paradigms. Closed-loop stimulation reflects this same principle, with multi-channel, open-source stimulators that record evoked responses and update parameters in real time, providing a potentially scalable model for dynamic recalibration based on cortical signals or behavioral feedback [113].

Convergence between neuroscience and information and communications technology is enabling wireless control, real-time processing, and bidirectional feedback in next-generation visual prostheses [114]. Progress in nanotechnology, gene therapy, and bioelectronic interfaces is enhancing electrode–tissue interfaces and intelligent stimulation. Hybrid approaches such as optogenetics allow for cell-type-specific activation with high spatial precision, but there remain challenges related to delivery [115]. Magnetic stimulation via nanoscale particles is being explored as a less invasive option. These trends signal a new direction toward long-term, responsive, and patient-specific vision implants. Through the incorporation of closed-loop algorithms, high-resolution arrays, and biologically tuned materials, prosthetics in the future may increasingly converge toward the restoration of functional, stable vision.

### Limitations and Extensions of Brain-Based Vision Interfaces

The concept of vision restoration without an intact eye has progressed from a theoretical concept to an area of research. As these technologies mature, they also raise fundamental questions about the scope, purpose, and limits of brain-based vision restoration beyond the eye. Earlier prostheses target the retina or the optic nerve, but cortical visual prostheses bypass ocular structures altogether by electrically stimulating the visual cortex to produce phosphenes. Although current cortical BCIs remain experimental and limited in resolution, recent advances have prompted discussion of their long-term conceptual implications. Niketeghad and Pouratian trace the history of cortical prostheses, such as Orion and Gennaris, which employ intracranial electrodes to generate spatially organized phosphenes [2]. While current systems possess limited spatial resolution and pattern recognition, they provide the potential of “sight without eyes.” Future devices may instead emphasize task-specific enhancement rather than attempting to reproduce natural vision [74]. A “smart bionic eye” can offer AI-enhanced overlays for navigation or object identification and function as a cognitive prosthesis that interprets the world instead of imitating biological vision.

Despite these advances, cortical BCIs face major challenges. Variability in phosphene perception, electrode interface instability, and the need for training in potential users limit the present use of BCIs [116]. fMRI and electrophysiology have confirmed that perception and visual imagination share overlapping neural pathways, such as the primary visual cortex, which is consistent with the proposed idea that BCI input can be learned and integrated [117]. In this framing, BCIs function not only as prosthetic substitutes for lost vision, but as neurotechnological interfaces that reshape how visual information is constructed, interpreted, and learned over time.

### Neural Vision Interfaces in Spaceflight Environments

**Fig. 6:**
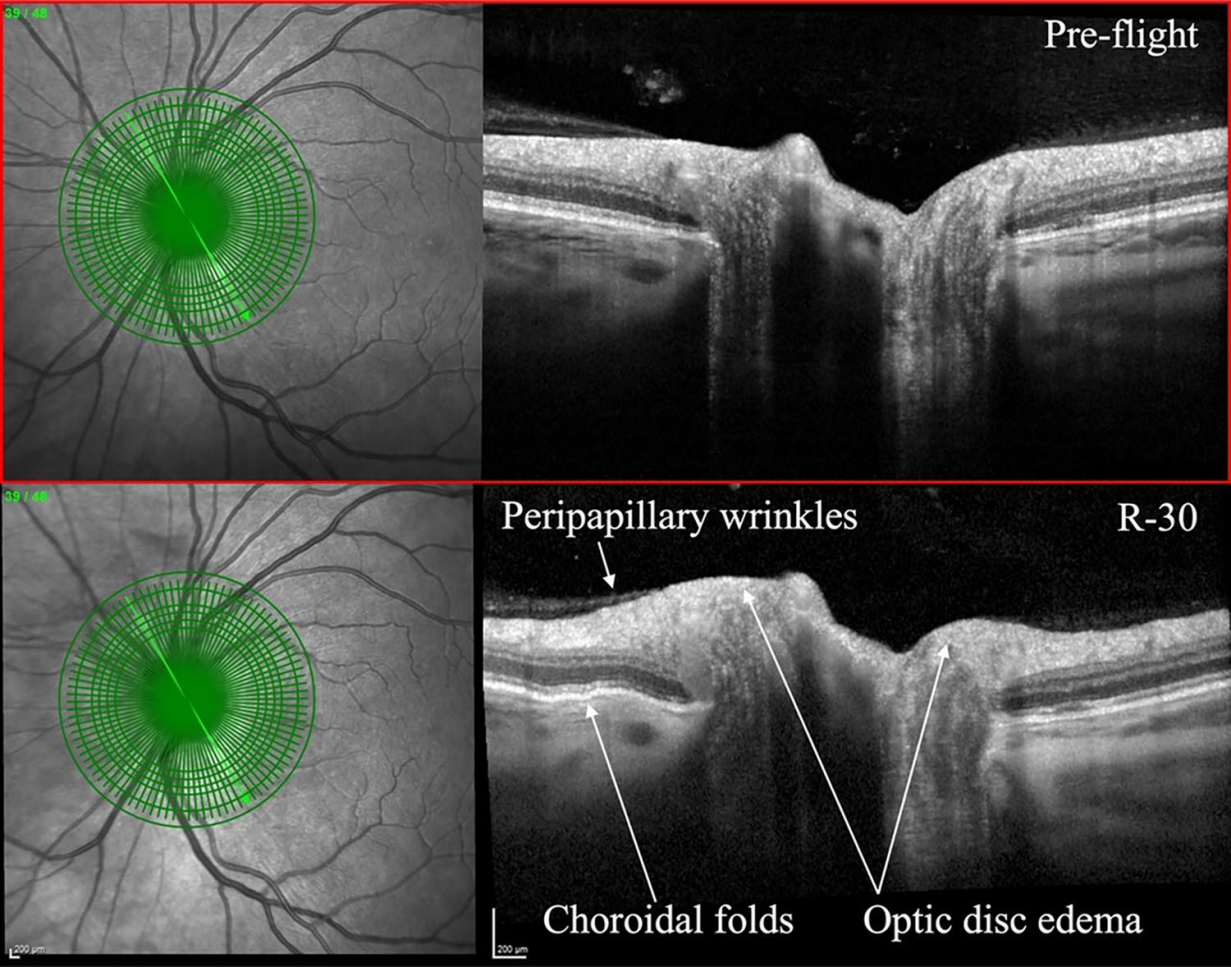
OCT images of the optic nerve head and peripapillary region. Pre-flight (top) and at R-30 (bottom), showing peripapillary wrinkles, choroidal folds, and optic disc edema consistent with spaceflight-associated neuro-ocular syndrome (SANS). Figure and legend reproduced from Ong et al., 2023 [119] under the Creative Commons Attribution NonCommercial (CC BY-NC 4.0) International License (https://creativecommons.org/licenses/by-nc/4.0/legalcode). PMID: 36690421

As neural vision implants move closer to clinical use, they are also being applied in the context of spaceflight. Spaceflight environments present physiological and operational challenges, including visual deprivation, altered sensorimotor integration, and limited access to in-flight clinical intervention. Spaceflight-associated neuro-ocular syndrome (SANS) affects astronauts on extended missions during space exploration, contributing to choroidal folds, optic disc edema, and visual disturbances (Fig. 6) [118, 119]. Because neural interfaces have the potential to restore spatial perception among blind individuals on Earth, there is growing interest in adjusting these systems to reverse blindness in space or improve sight in hazardous, low-light environments.

The implications of improvements in subretinal and cortical implants in terms of resolution, wireless function, and biocompatibility suggest that such systems can be used in extreme settings, such as space or disaster zones [120]. Subretinal photovoltaic implants are now capable of being implanted *in situ* with higher-resolution arrays without damage to the retina, a significant consideration for longer missions or astronauts with early-stage SANS [112]. At the same time, low-light image enhancement can be performed via deep-learning technology that uses convolutional neural networks and generative adversarial networks. Improving images under low-light settings can reduce visual noise and improve visibility in degraded environments [5]. BCIs can also influence visual gaze abnormalities in spaceflight, such as increases in gaze latency, which occur as a result of the decreased saccadic velocity and head movement [121, 122]. Closed-loop, modular stimulatory systems with real-time adaptability are currently under development, and they aim to combine wearable AI and multisensory input to respond to both environmental and behavioral feedback [113, 121].

Building on earlier concepts of prosthetic vision, many researchers envision “smart bionic eyes” aimed at task-specific improvement. Instead of replicating natural vision, these systems could deliver overlays specific to navigation, hazard detection, or facial recognition [74]. Virtual reality-based training and user feedback can be utilized to guide pre-mission adaptation by astronauts or help first responders in dynamic settings. As these technologies evolve from being therapeutic devices to real-time vision augmentation systems, they have an increasingly realistic potential to improve performance in space exploration, field operations, and emergency responses.

## Conclusion

Recovery of vision via neural interfaces is a potentially transformative intersection of neuroscience, engineering, and clinical care. Researchers have significantly improved the ability to encode artificial signals into percepts that the brain can interpret. However, artificial vision is still a unique modality, one which must be learned cognitively and supported by neuroplastic adaptation and rehabilitation. Advances in machine learning and AI may eventually allow for adaptive stimulation strategies, closed-loop feedback, and dynamic interpretation of scenes, matched to neural activity in individual patients to improve clinical viability. Transcranial stimulation and ultrasound offer non-invasive alternative mechanisms for activating the visual system. All of these innovations extend the utility of neural vision technologies beyond traditional blindness into low-vision rehabilitation, disaster response, and space exploration. Ultimately, the restoration of vision represents a systems-level endeavor that requires interdisciplinary collaboration and integration within the user’s perceptual and psychological framework. Rather than replicating natural vision, these technologies aim to construct a new visual language, one that allows humans to navigate the world via redefined sensory terms.

## Data Availability

This is a literature-based review. All data supporting the findings of this study are derived from published sources, which are cited throughout the manuscript and available via public databases or journals.

## Abbreviations

EEG: Electroencephalogram
RP: Retinitis Pigmentosa
BCIs: Brain-Computer Interfaces
AI: Artificial Intelligence
AMD: Age-Related Macular Degeneration
RGCs: Retinal Ganglion Cells
FDA: Food and Drug Administration
CE: Conformité-Européenne
RPE: Retinal Pigment Epithelium
AR: Augmented Reality
NIR: Near-Infrared
AV-DONE: Artificial Vision by Direct Optic Nerve Electrode
LGN: Lateral Geniculate Nucleus
TMS: Transcranial Magnetic Stimulation
tDCS: Transcranial Direct Current Stimulation
TOPhos: Task Optimized Phosphene Vision
fMRI: Functional Magnetic Resonance Imaging
ECoG: Electrocorticography
SANS: Spaceflight-Associated Neuro-Ocular Syndrome
